# Effects and limitations of home-based motor-control exercise for chronic low back pain: A single center prospective study

**DOI:** 10.1371/journal.pone.0284741

**Published:** 2023-04-24

**Authors:** Ryosuke Hirota, Atsushi Teramoto, Takanori Murakami, Mitsunori Yoshimoto, Noriyuki Iesato, Toshihiko Yamashita

**Affiliations:** 1 Department of Orthopedic Surgery, Sapporo Medical University School of Medicine, Sapporo, Japan; 2 Division of Rehabilitation Medicine, Sapporo Medical University School of Medicine, Sapporo, Japan; Pozan University of Physical Education, POLAND

## Abstract

**Study design:**

Prospective single-center observational study.

**Objective:**

To investigate the effects and limitations of self-motor-control exercise in patients with chronic low back pain.

**Summary of background data:**

Although exercise therapy and physical therapy have been shown to be effective in treating chronic low back pain, these therapies are often discontinued due to patients’ non-compliance, and their effectiveness cannot be fully demonstrated.

**Methods:**

Fifteen patients with low back pain, no apparent organic disease, who had been symptomatic for at least three months, and could continue motor-control exercise at home for at least six months were included in the study. Low back pain (visual analog scale [VAS]), locomotor 25, stand-up test, two-step test, trunk and total body muscle mass by the impedance method, and spinal sagittal alignment were examined before the intervention to establish a baseline, and at two and six months after the intervention.

**Result:**

Significant improvement was observed in the back pain VAS (p<0.01), stand-up test (p = 0.03), two-step test (p = 0.01), and locomotor 25 (p = 0.04) before and after the intervention. In contrast, there were no significant changes in muscle mass and sagittal alignment. The effect of long-term exercise was more pronounced in patients without spinal deformity.

**Conclusions:**

Self-exercise for patients with chronic low back pain was effective in improving pain and function, although it did not directly affect muscle mass or alignment. Moreover, strength training of the lumbar back muscles alone was not found to be effective in patients with spinal deformities.

## Introduction

Chronic low back pain is defined as low back pain that persists longer than three months [1]. Patients with acute low back pain typically show marked improvement in pain within six weeks of its onset, followed by gradual improvement of up to 52 weeks. On the contrary, the pain scores of patients with chronic low back pain show improvement at six weeks, but the pain persists thereafter [2]. It is estimated that more than 25 million people in Japan have low back pain, and the associated medical and economic burden is enormous [3]. Pharmacotherapy, including non-steroidal anti-inflammatory drugs [4, 5], serotonin and norepinephrine reuptake inhibitors [6], and weak opioids [7], have been reported useful for treating chronic low back pain.

Exercise therapy is also known to be helpful treating chronic low back pain. It is associated with the prevention and improvement of low back pain, and there is a known correlation between an active daily lifestyle and prevention of chronic low back pain. Therefore, a lifestyle that incorporates a moderate degree of exercise is recommended. In a randomized controlled trial of patients with chronic low back pain, exercise therapy was found to be effective in improving the range of motion in individuals with low back pain, as well as improving the functional disability, pain, motor function, health status, muscle strength, and endurance [8]. Exercise therapy also improves the quality of life in patients with chronic low back pain [9]. A comparison of an exercise and an oral group taking non-steroidal anti-inflammatory drugs showed that the low back pain-related quality of life was significantly improved in the exercise group [10]. In addition, no complications or cranial events have been reported with exercise therapy [11], rendering exercise therapy a safe and useful treatment method. At present, however, there are no reports that clearly indicate which type of exercise therapy is most effective, and its long-term effects are not yet clear.

Motor-control exercise involves training to improve the muscle function of the deep extensor muscles of the trunk, such as the transversus abdominis, internal abdominal oblique, and multifidus muscles, with the aim of improving spinal stability. Compared to a control group, improvements in pain and physical function have been observed through motor-control exercise training [12]. Training methods can be adopted to incorporate weights appropriate for the age and functional ability of the patient and should be performed relatively easily. Ideally, exercise therapy should be implemented under the guidance of a specialist, however continuous visits to the hospital are not practical; therefore, it is important to encourage patients to continue exercising at home.

The purpose of this study was therefore to determine the effectiveness and limitations of performing motor-control exercises at home in patients with chronic low back pain.

## Materials and methods

### Study design

This is a single-center prospective observational study.

### Ethics

**Fig 1:**
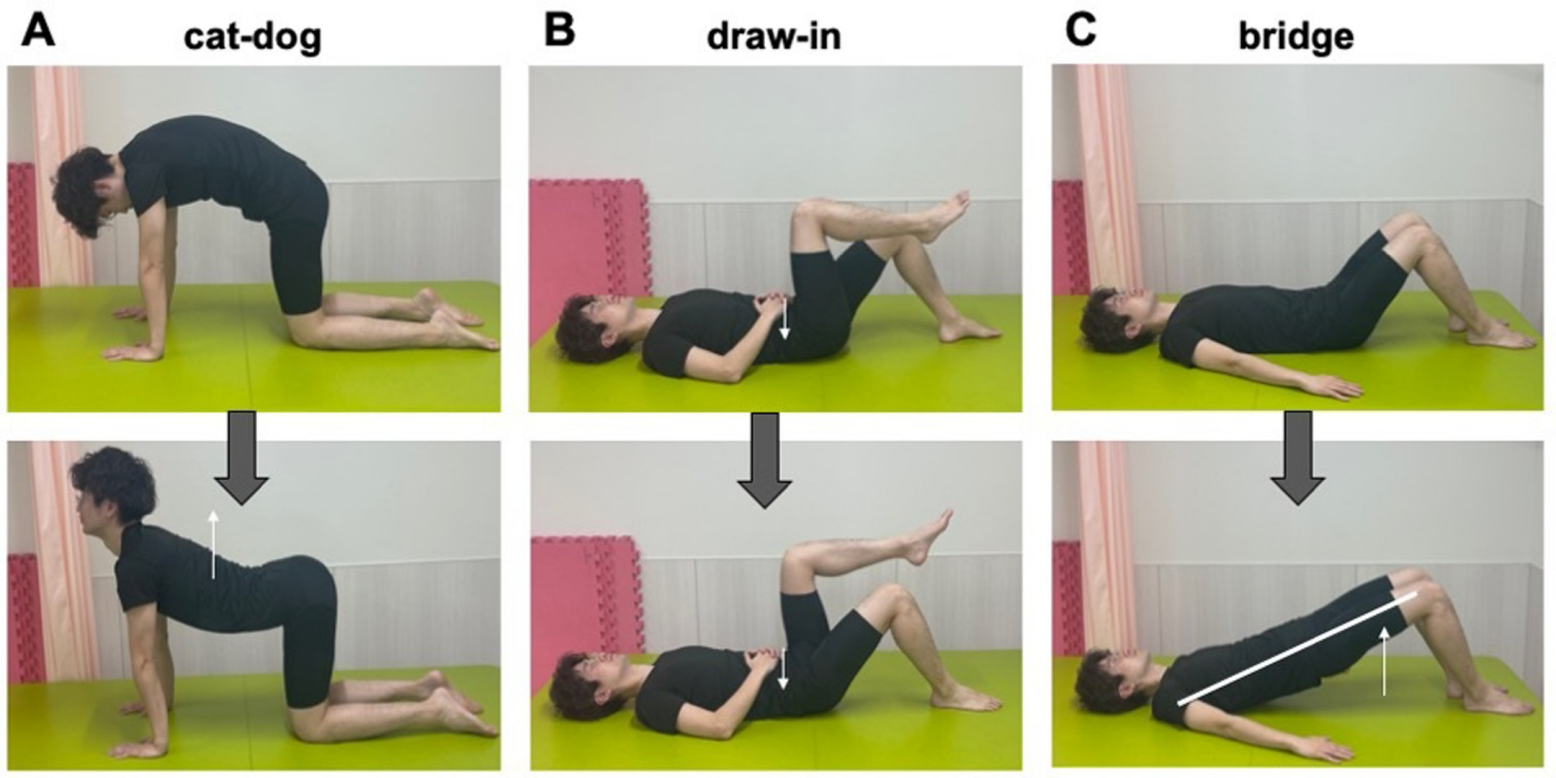
Schematic diagram of each self-motor-control exercise. A) Cat-dog. B) Draw-in. C) Bridge.

The institutional review board of the representative facility reviewed and approved this study (Sapporo Medical University, No.292-182). Informed consent documents obtained from all participants in this study are availbale for publication (as outlined in PLOS consent form). Further, the study participant provided written informed consent (as outlined in the PLOS consent form) to publish the case details, including images shown in Fig 1.

### Patient population

Fifteen patients with low back pain, no apparent organic disease, had been symptomatic for at least three months, and could continue motor-control exercise at home for at least six months were included in the study (average age 60.4 years). In addition, based on the Scoliosis Research Society–Schwab adult spinal deformity (ASD) classification [13], four patients were classified into the ASD group and 11 into the non-ASD group. The ASD group comprised of patients with pelvic incidence–lumbar lordosis (PI–LL) of >20°, sagittal vertical axis (SVA) of >9.5 cm, or pelvic tilt (PT) of >30° on lateral radiographs before the intervention.

Patients with a history of surgery, cases in which the back pain was caused by malignancies, fractures, or infections, and cases in which back pain was of visceral origin, such as of the renal–urinary system or due to gynecological disease, were excluded. Baseline testing, prior to intervention, confirmed that the stand-up and locomotor 25 tests yielded significantly worse motor function results for the ASD group when compared to the non-ASD group. In contrast, there were no significant differences in muscles mass or the degree of back pain between the two groups (Table 1).

**Table 1:** General characteristics of all patients.

	ASD (n = 4)	Non-ASD (n = 11)	*p*
Sex (female %)	100	81.8	0.45
Age (years)	68.4±12.4	56.5±17.2	0.04
Sagittal alignment			
SVA (mm)	119.3±46.1	0.68±16.4	<0.001
PT (°)	29.0±9.20	15.0±4.15	0.02
PI–LL (°)	28.6±11.3	1.23±6.65	<0.001
TPA (°)	35.4±7.87	19.8±4.98	0.01
Motor function			
Standing test	2.0±1.29	3.83±0.96	0.001
Two-step test	1.04±0.27	1.19±0.18	0.23
Locomotion 25	54.2±18.3	21.5±8.86	0.003
Back pain VAS	7.60±0.45	7.14±0.89	0.69
Muscle mass			
Total muscle mass (kg)	39.0±4.15	39.1±7.19	0.90
Trunk muscle mass (kg)	16.7±2.76	15.5±3.23	0.78

ASD, adult spinal deformity; SVA, sagittal vertical axis; PT, pelvic tilt; PI–LL, pelvic incidence–lumbar lordosis; TPA, T1 pelvic angle; VAS, visual analog scale.

### Motor-control exercise

The intervention exercises were the following: “cat-dog” as a trunk flexion exercise, “bridge” as an extension exercise, and “draw-in” for deep muscle strengthening (Fig 1). In cat-dog, the patient went down on their hands and knees in a quadruped position and repeatedly performed a combined posterior pelvic tilt and lumbar kyphosis movement. In draw-in, patient went into a supine position and repeatedly exhaled fully with simultaneous maximum abdominal contraction. In bridge, patient remained in a supine position, flexed the knee joints with feet planted, and extended the hip joints by elevating the torso. The exercises were performed with a volume and intensity that did not aggravate the back pain.

The patients were instructed on content and intensity of the exercises during the rehabilitation sessions at the clinic. The patients were instructed to exercise at least twice weekly for 20 minutes a day for six months.

### Evaluation items

The low back pain (Visual Analog Scale, [VAS]), locomotor 25 [14], stand-up test [14], two-step test [14], trunk and total body muscle mass by the impedance method (InBody M20, InBody Japan Inc., Tokyo, Japan), and spinal sagittal alignment were examined before the intervention, and at two and six months after the start of the intervention.

### Statistical analysis

Continuous variables were evaluated using the Student’s and paired *t*-tests. Non-continuous variables were evaluated using the Fisher’s exact test. To compare multiple groups, one-way analysis of variance and the Bonferroni post-hoc testing were employed. A *p*-value of <0.05 denoted statistical significance. All statistical analyses were conducted with SPSS version 25.0 (IBM Corporation, Armonk, NY, USA).

### Ethics

The present study was conducted in accordance with the Helsinki Declaration, and the patients provided informed consent prior to participation. The study protocol was approved by the institutional review boards of all participating hospitals.

## Results

### Pain

**Fig 2:**
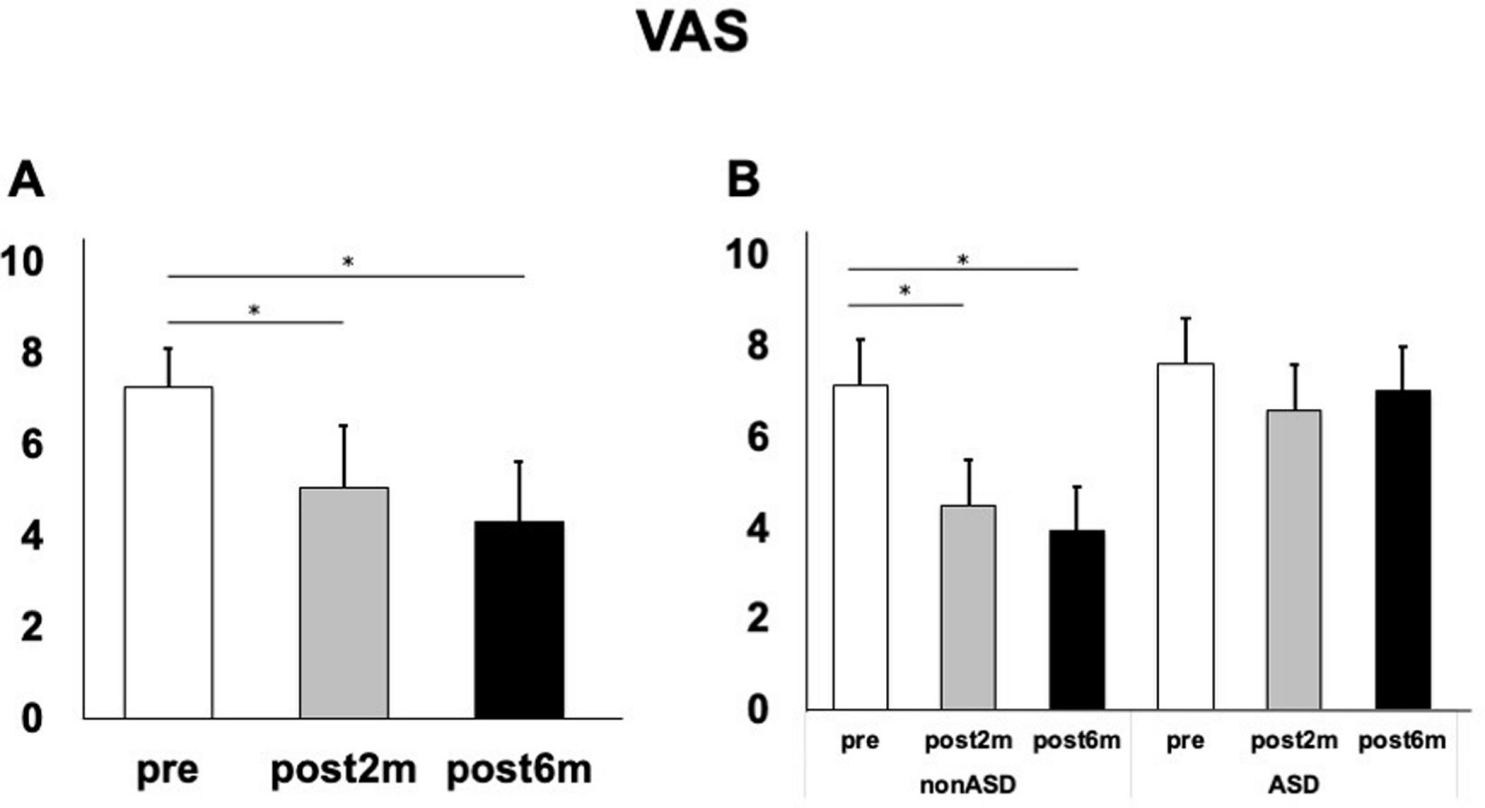
Low back pain visual analog scale before and after the intervention. A) Changes in low back pain in all patients. B) Changes in low back pain in the adult spinal deformity (ASD) and non-ASD groups, respectively.

The low back pain (VAS) improved as a function of time between pre-intervention and six months post-intervention [(7.26 vs 4.33 p = 0.01 (Fig 2A)]; however, the improvement was significant only in the non-ASD group (7.14 vs 3.92 p = 0.01) and not in the ASD group (Fig 2B).

### Function

**Fig 3:**
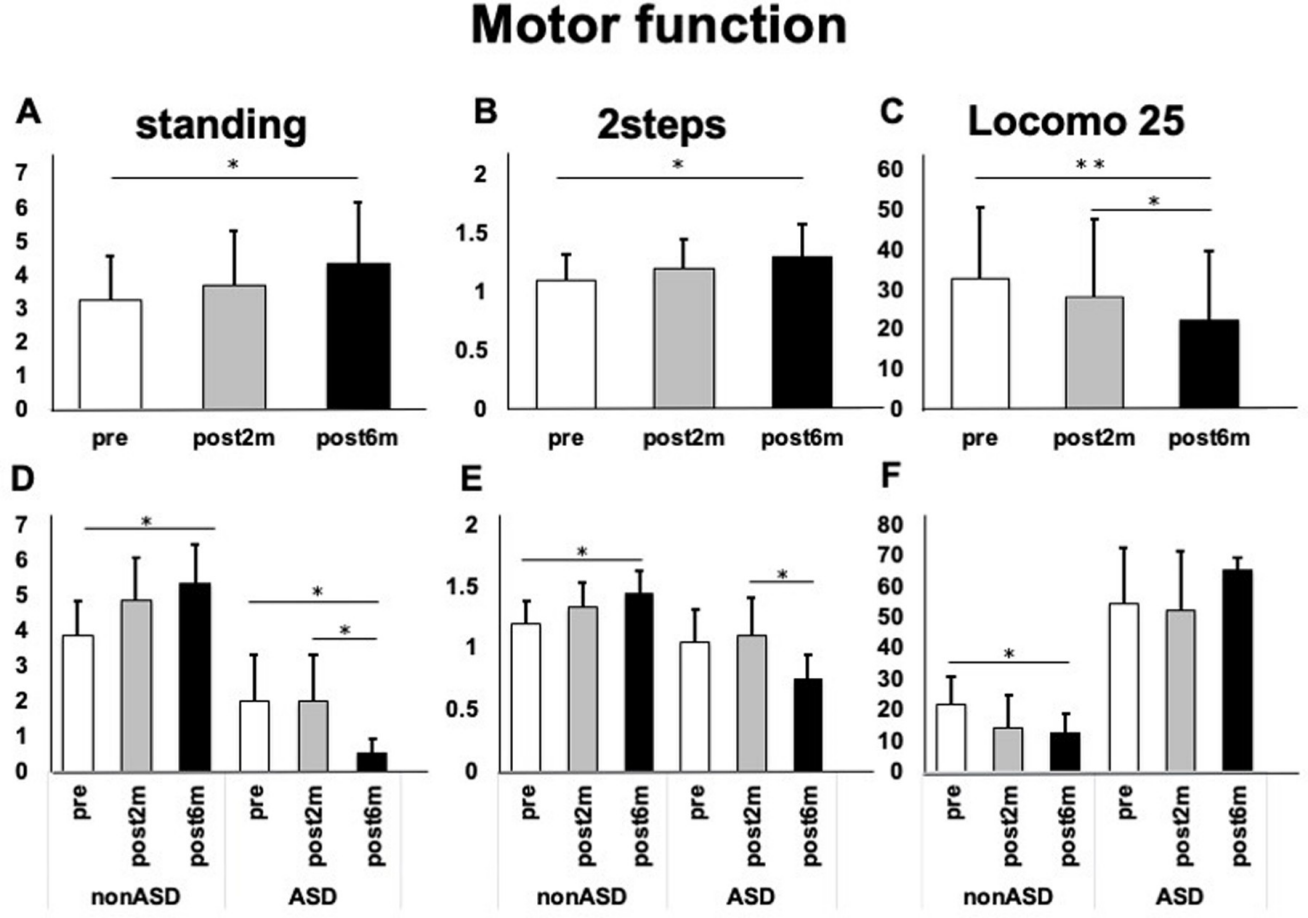
Motor function before and after intervention. A) Changes in the standing test in all patients. B) Changes in the two-step test in all patients. C) Changes in the locomotor 25 test in all patients. D) Changes in the standing test in the ASD and non-ASD groups. E) Changes in the two-step test in the ASD and non-ASD groups. F) Changes in the locomotor 25 test in the ASD and non-ASD groups (*p<0.05).

Various functional tests showed improvement over time with significant differences between pre-intervention and six months post-intervention (standing test; 3.22 vs 4.33 p = 0.03, 2 steps test; 1.10 vs 1.30 p = 0.04, locomo25; 32.6 vs 22.2 p = 0.01) (Fig 3A–3C). The non-ASD group showed improvement as a function of time after the intervention (standing test; 3.83 vs 5.33 p = 0.01, 2 steps test; 1.19 vs 1.43 p = 0.04, locomo25; 21.5 vs 12.5 p = 0.02), whereas the ASD group showed no improvement (Fig 3D–3F).

### Muscle volume

**Fig 4:**
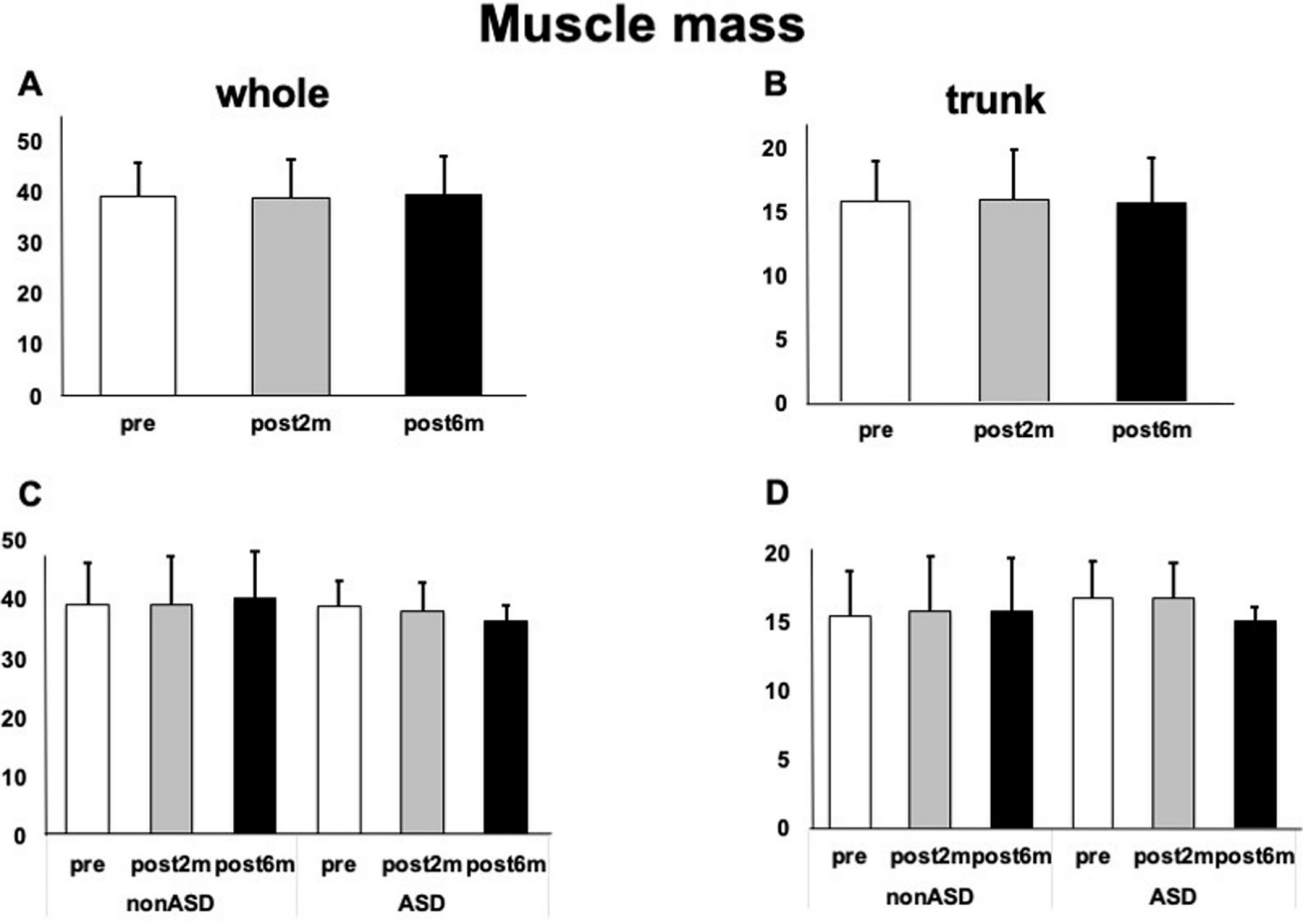
Muscle mass before and after intervention. A) Total muscle mass change in all cases. B) Changes in trunk muscle mass in all cases. C) Changes in total muscle mass in the ASD and non-ASD groups. D) Changes in trunk muscle mass in the ASD and non-ASD groups.

There was no significant change in the muscle volume in the ASD and non-ASD groups (Fig 4).

### Spinal alignment

**Fig 5:**
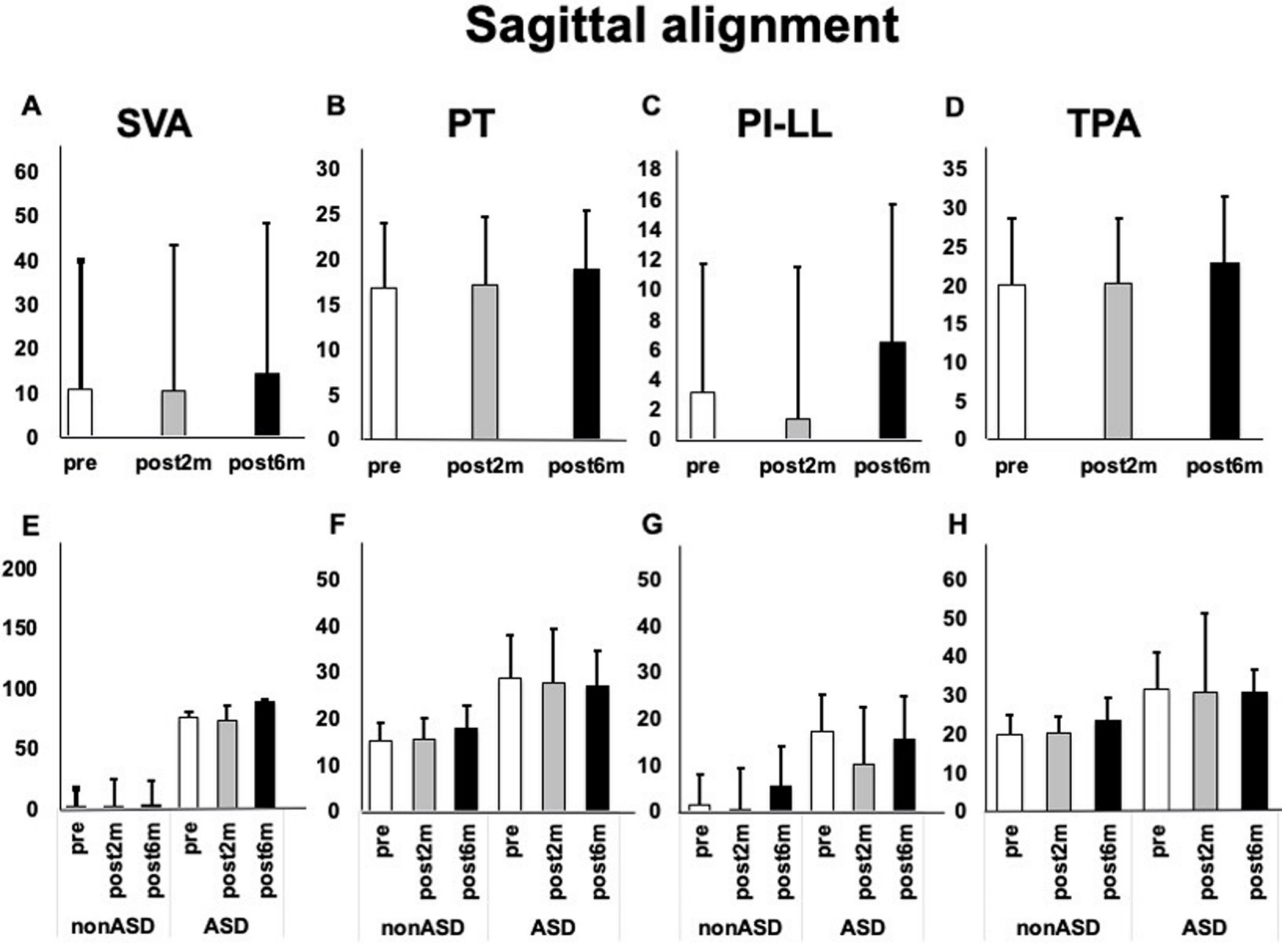
Sagittal alignment before and after intervention. A) Changes in the sagittal-vertical axis (SVA) in all patients. B) Changes in the pelvic tilt (PT) in all cases. C) Changes in the pelvic incidence–lumbar lordosis (PI–LL) in all patients. D) Changes in the T1 pelvic angle (TPA) in all patients. E) Changes in the SVA in the ASD and non-ASD groups. F) Changes in the PT in the ASD and non-ASD groups. G) Changes in the PI–LL in the ASD and non-ASD groups. H) Changes in the TPA in the ASD and non-ASD groups.

There was no significant change in the spinal sagittal alignment in the ASD and non-ASD groups (Fig 5).

## Discussion

In this study, self-exercise was shown to improve motor function and pain in patients with chronic low back pain, although spinal alignment and muscle mass did not improve. There are several hypotheses that could explain how exercise facilitates these improvements. First, exercise therapy may have helped relieve nociceptive or neuropathic pain by gradually stretching immobilized tissues and restoring structural and functional mechanisms including alignment, blood flow, and oxygen uptake. Second, the strengthening of paraspinal muscles may contribute to pain relief by stabilizing the areas of nociceptive pain caused by mechanical instability. Third, exercise tends to increase endogenous opioid secretion, contributing to the activation of the descending pain suppression system [15]. Continuous exercise habits may also result in pain relief via the central pain modification system. Additionally, exercise may have contributed to the relief of low back pain by suppressing minor chronic inflammation caused by inactivity and obesity, which are common in patients with chronic low back pain, through expression and activation of the transcription factor PGC1α [16].

Patients without spinal deformity showed improvement with prolonged exercise, but no effect was observed in patients with spinal deformity. There is a significant correlation between abnormal spinal sagittal alignment and health-related quality of life scores [17]. Progressive kyphosis deformity of the spine leads to poor balance and low back pain from fatigue in the standing position. In addition, as lumbar kyphosis decreases, isometric contraction occurs with the dorsal muscles elongated, this in turn increases mechanical stress on the intervertebral discs. Moreover, continuous contraction of the back muscles to maintain balance is thought to reduce blood flow to the muscles and increase internal pressure, resulting in muscular low back pain [18, 19].

The significance of exercise therapy for adult spinal deformity remains unclear. There is sufficient evidence that the progression of deformity is associated with degeneration of the intervertebral discs as well as weakness of the back muscles. Therefore, exercise therapy may be effective in preventing the progression of spinal deformity. However, in this study, trunk muscle training alone did not improve pain or motor function in cases of spinal deformity.

A previous report has shown the effectiveness of stretching and strength training interventions in improving the flexibility of the lower extremities, such as the hip joint, as well as the trunk, in the treating the spinal deformity [20]; however, interventions in the proximal muscles of the lower extremities, such as the gluteus maximus and medius, may also be useful.

Nevertheless, the long-term effects of exercise therapy on low back pain have not been clearly demonstrated. While some reports indicate that the effects of pain relief and improvement in functional disability are maintained 12 months after the end of treatment [21], others demonstrate no benefit in long-term intervention [22].

In the present study, exercise improved motor function and pain over a longer period of time, especially in patients without spinal deformity. Exercise has been shown to be effective in preventing new or recurrent low back pain [23], and we believe it should be continued in patients with low back pain without spinal deformity provided no adverse events occur.

There are several limitations to this study. First, the sample size was small. Second, the intervention period was limited to six months. Third, oral medications were not investigated. Nevertheless, the effects of self-motor-control exercise from multiple perspectives, including pain, motor function, muscle mass, and sagittal alignment have not been previously prospectively investigated. Self-motor-control exercise at home is extremely useful for alleviating pain and restoring motor functions in patients with chronic low back pain, in the absence of spinal deformity. In future, it would be desirable to examine in more detail how varying the intensity of self-motor control exercises and the duration of intervention affect chronic low back pain.

## Supporting information

S1 Data (XLSX)
